# Real-time breath recognition by movies from a small drone landing on victim’s bodies

**DOI:** 10.1038/s41598-021-84575-1

**Published:** 2021-03-03

**Authors:** Takeji Saitoh, Yoshiaki Takahashi, Hisae Minami, Yukako Nakashima, Shuhei Aramaki, Yuki Mihara, Takamasa Iwakura, Keiichi Odagiri, Yuichiro Maekawa, Atsuto Yoshino

**Affiliations:** 1 Department of Emergency and Disaster Medicine, Hamamatsu University School of Medicine, Hamamatsu, 431-3125 Japan; 2 Center for Clinical Research, Hamamatsu University Hospital, Hamamatsu, 431-3125 Japan; 3 Department of Cardiology, Hamamatsu University School of Medicine, Hamamatsu, 431-3125 Japan

**Keywords:** Medical research, Signs and symptoms

## Abstract

In local and global disaster scenes, rapid recognition of victims’ breathing is vital. It is unclear whether the footage transmitted from small drones can enable medical providers to detect breathing. This study investigated the ability of small drones to evaluate breathing correctly after landing on victims’ bodies and hovering over them. We enrolled 46 medical workers in this prospective, randomized, crossover study. The participants were provided with envelopes, from which they were asked to pull four notes sequentially and follow the written instructions (“breathing” and “no breathing”). After they lied on the ground in the supine position, a drone was landed on their abdomen, subsequently hovering over them. Two evaluators were asked to determine whether the participant had followed the “breathing” or “no breathing” instruction based on the real-time footage transmitted from the drone camera. The same experiment was performed while the participant was in the prone position. If both evaluators were able to determine the participant’s breathing status correctly, the results were tagged as “correct.” All experiments were successfully performed. Breathing was correctly determined in all 46 participants (100%) when the drone was landed on the abdomen and in 19 participants when the drone hovered over them while they were in the supine position (p < 0.01). In the prone position, breathing was correctly determined in 44 participants when the drone was landed on the abdomen and in 10 participants when it was kept hovering over them (p < 0.01). Notably, breathing status was misinterpreted as “no breathing” in 8 out of 27 (29.6%) participants lying in the supine position and 13 out of 36 (36.1%) participants lying in the prone position when the drone was kept hovering over them. The landing points seemed wider laterally when the participants were in the supine position than when they were in the prone position. Breathing status was more reliably determined when a small drone was landed on an individual’s body than when it hovered over them.

## Introduction

When there is a chemical, biological, radiological, nuclear, and high explosive (CBRNE) event, rapid identification and initial management of exposed victims is vital^1^. In such situations, responders should wear personal protective equipment, enter the hot zone, and evaluate casualties. However, impassable roads and pathways may block access to disaster scenes. Despite this, medical providers need to take the risk and approach the victims to implement triage protocols. Prehospital triage protocols have been studied extensively over the past decades^2–4^. Breathing must be evaluated as one of the early steps of triage assessment. Victims with apnea are given low priority because they consume a disproportionate share of resources. To address these issues, simple, speedy, safe, and cost-effective methods must be used when handling victims in those problematic circumstances. The use of unmanned aerial vehicles, known as drones, has demonstrated an unprecedented level of improvement in the efforts to search for survivors in the aftermath of disasters^5,6^. Nowadays, a drone equipped with a camera can reportedly evaluatehuman breathing using lasers^7^. However, drones cannot be used in small spaces and are not entirely reliable. This study investigates the ability of a small cost-effective drone to evaluate breathing status correctly when it lands on a victim’s body and when it hovers over them.

## Methods

### Study design and participants

This was a prospective, randomized, crossover study, conducted between June and September 2020. Healthy participants aged 20 years or older, who were licensed Japanese medical doctors or nurses, were invited to participate in the experiment. Candidates with psychiatric disorders were excluded. Two medical doctors certified by the Japanese Society of Emergency Medicine were chosen to evaluate participants’ breathing. The participants and evaluators were blinded to the study design, aim, and endpoint. The drone pilot had fully practiced controlling using manikins for resuscitation training. The study was conducted in accordance with the Declaration of Helsinki, and the protocol was approved by the Hamamatsu University Ethics Committee (reference: 19-329). Written informed consent was obtained from all participants. The protocol was uploaded to the UMIN system (#UMIN: 000040702). Before the experiment, a preliminary study was performed on 10 healthy people: a drone was landed on their bodies and also hovered over them. The evaluators correctly judged the breathing status in 8 and 5 participants when the drone was landed on their bodies and when it hovered over them, respectively. We prospectively enrolled 46 participants as per the required sample size (α = 0.05, power = 0.8).

### Study protocol and methods of measurement

**Figure 1:**
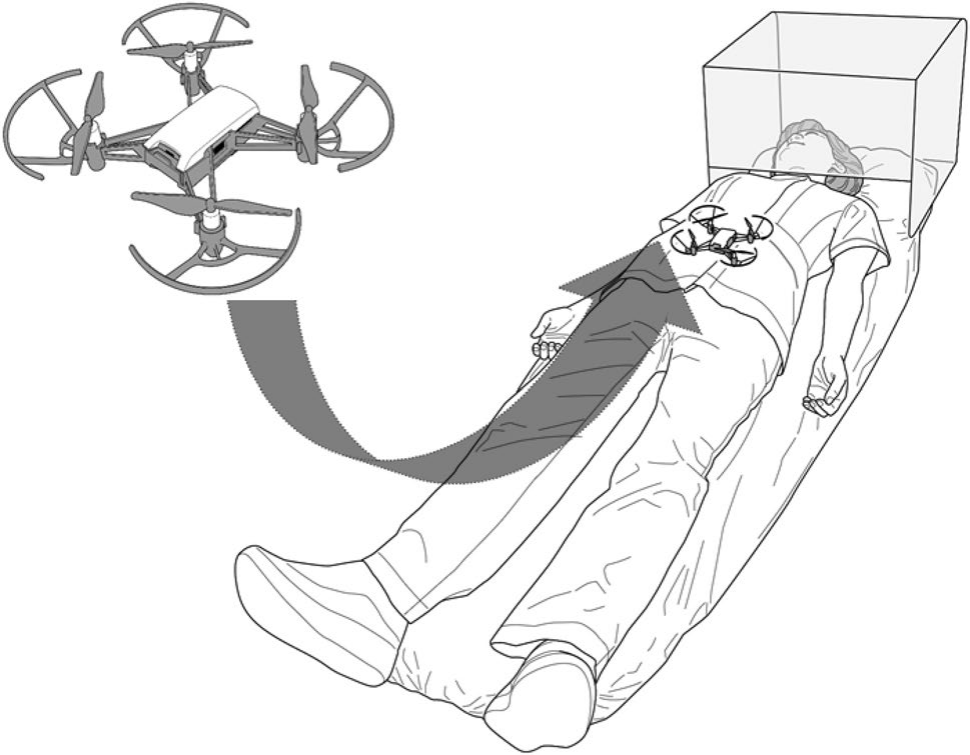
Schema of the drone landing on a participant’s abdomen.

We determined the participants’ height, weight, abdominal width (the distance between the lateral sides of the abdomen at the level of the umbilicus), and abdominal height (the distance between the tip of the xiphoid process and the pubic symphysis). A mattress (Transfermattress, Paramount Ltd., Tokyo, Japan) was placed on the floor in a confined room (depth × width × height: 3.6 × 3.4 × 2.8 m) with a brightness level of 500 lx. The participants were given instructions in four envelopes. They were to pull out four notes written either “breathing” or “no breathing” from each of the four envelopes. Each envelope contained 46 notes with each of the instructions (23 × 2). Participants who pulled out the “breathing” note were instructed to breathe ordinarily once every 3 s for 15 s; those who pulled out the “no breathing” note were instructed to hold their breath for 15 s. Participants were asked to lie on the mattress and were covered using an acrylic case (500 × 400 × 500 mm) over the head for their safety (Fig. 1). They watched the clock on the acrylic case for the timing of breathing. A small piece of colored tape was placed on their umbilicus as a target in this study.

First, the quadcopter-type drone (Tello, DJI Ltd., Shenzhen, China) with propeller guards was switched on, and the controller (GameSir T1d Controller, DJI Ltd., Shenzhen, China) was connected using a smartphone (iPhone 11.2.6., Apple Ltd., CA, USA) via Bluetooth. A double-sided adhesive tape (#775, Teraoka Seisakusho Ltd., Tokyo, Japan) was attached to the skids of the drone, and the drone was placed on a plastic launch pad (150 × 42 × 12 mm). A small drone (98 × 92.5 × 41 mm, 80 g) was used; it was equipped with an Intel 14-core processor, a front camera (field of view: 82.6°), and a pressure sensor. A collision detection system was placed 20 cm under the drone. The drone automatically hovered in the same position when pilots were not touching the sticks of the controller. The pilot and the two evaluators were kept out of the experiment room, with the door closed. They were only able to see the drone flying through the smartphone screen. In the first experiment, the participants were placed in the supine position. The pilot flew the drone off the launch pad in the room and landed it on the participants’ umbilicus, relying on the footage the smartphone transmitted from the camera in real-time. After touchdown, the administrator called out the start of the orders determined by the notes to a participant when the drone set the position. The two evaluators carefully examined the footage transmitted from the drone-mounted camera for chest movement in real-time and independently evaluated the participants’ breathing status. The evaluators assessed the participants’ breathing for 10 s^8^ and answered either “breathing” or “no breathing.” The two evaluators’ responses were tagged as “correct” when both of them answered correctly; otherwise, they were tagged as “incorrect.” “Incorrect” responses were grouped into either “completely incorrect” or “undetermined.” “Completely incorrect” responses indicated that the two evaluators provided the same incorrect answer. “Undetermined” indicated that the two evaluators provided different answers. In the second experiment, the drone was flown from the caudal side of the participants’ body and was kept hovering approximately 20 cm above the participants’ body (closer to the chest and abdomen), allowing the evaluators to observe the participant’s breathing. Chest movement was evaluated in a way similar to that in the first experiment. In the third experiment, the participants were placed in the prone position. The drone landed on the body point that corresponds to the location of the umbilicus. Again, the participants’ breathing was evaluated. In the fourth experiment, the pilot kept the drone hovering over the participants’ body, and the participants’ breathing status was determined. After landing the drone on the participants’ bodies positioned in the supine and prone positions, we took a photograph of each participant with the drone and a scaler placed on them. The pictures were saved on a personal computer and the distance from the umbilicus to the center of the drone was measured. Furthermore, the angle of the drone’s nose was measured based on the cranial-caudal axis. The primary goal was to determine the accuracy of evaluating breathing status when the drone was landed and kept hovering.

### Statistical analysis

The data were recorded and analyzed in Microsoft Excel 2016 (Microsoft Corporation, Redmond, WA, USA). Continuous data were expressed as means ± standard deviations (SD) or medians with interquartile ranges (IQR). The Kolmogorov–Smirnov test was used to evaluate the normality of distribution. For data with a parametric distribution, a two-sided paired t-test was used to compare two continuous variables. Categorical data were analyzed using the chi-square test, and accuracy was analyzed using McNemar’s analysis.

## Results

### Participants

We enrolled 46 participants consisting of 23 men and 23 women. None of the participants were excluded. The median age of the participants was 27 years (IQR: 24–33 years, minimum to maximum: 22–63 years). Their average height, body weight, body mass index, abdominal width, and abdominal height were 165 ± 9 cm, 59 ± 11 kg, 21.4 ± 2.8 kg/m^2^, 28 ± 4 cm, and 34 ± 3 cm, respectively. All participants completed the experiments without experiencing any adverse events.

### Accuracy of evaluating breathing

Breathing was evaluated more reliably when the drone was landed on the front and back than when it was kept hovering over the participants’ body (Table 1). When the drone was landed on the front, none of the evaluators provided incorrect answers. When the drone was kept hovering over the participants’ body, the evaluators incorrectly determined the breathing status for half of the participants in both the supine and prone positions.

**Table 1:** Judgment of breathing.

	Hovering	
	Correct	Incorrect
**Supine, N = 46**		
Landed on front		
Correct	19	27
Incorrect	0	0
**Prone, N = 46**		
Landed on back		
Correct	10	34
Incorrect	0	2

McNemar’s analyses were performed (p < 0.01, respectively).

### Correct answer during breathing and no breathing assessments

When we compared correct responses for the two categories (“breathing” and “no breathing”), there were fewer correct answers when the drone was kept hovering than when it was landed, as shown in Table 2.

**Table 2:** Classification of the correct judgment of breathing.

Supine	Landed on front	Hovering	p
Breathing	23/23 (100%)	8/23 (34.7%)	< 0.01
No breathing	23/23 (100%)	11/23 (47.8%)	< 0.01

Prone	Landed on back	Hovering	p
Breathing	22/23 (95.6%)	2/23 (8.6%)	< 0.01
No breathing	22/23 (95.6%)	8/23 (34.7%)	< 0.01

Chi-square analyses were performed.

### Incorrect responses for when the drone was kept hovering

**Figure 2:**
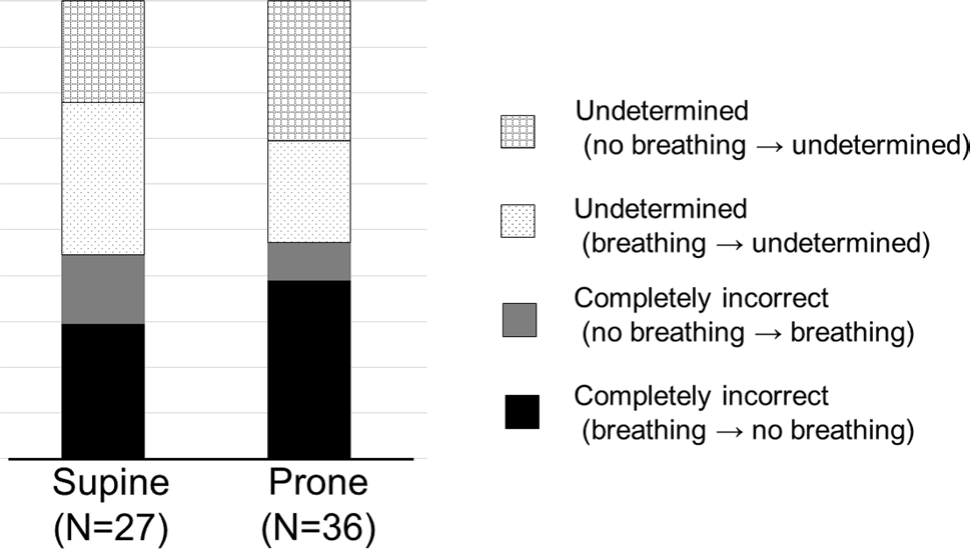
Incorrect responses for when the drone was kept hovering on the participants.

The ratio of the groups in incorrect answers did not differ according to participants’ positions when the drone was kept hovering, as presented in Fig. 2. Instances where “breathing” was stated as “no breathing” (completely incorrect, false negative) accounted for 8/27 (29.6%) and 13/36 (36.1%) of the answers in the supine and prone position, respectively, when the drone was kept hovering.

### Landing points of the drone

**Figure 3:**
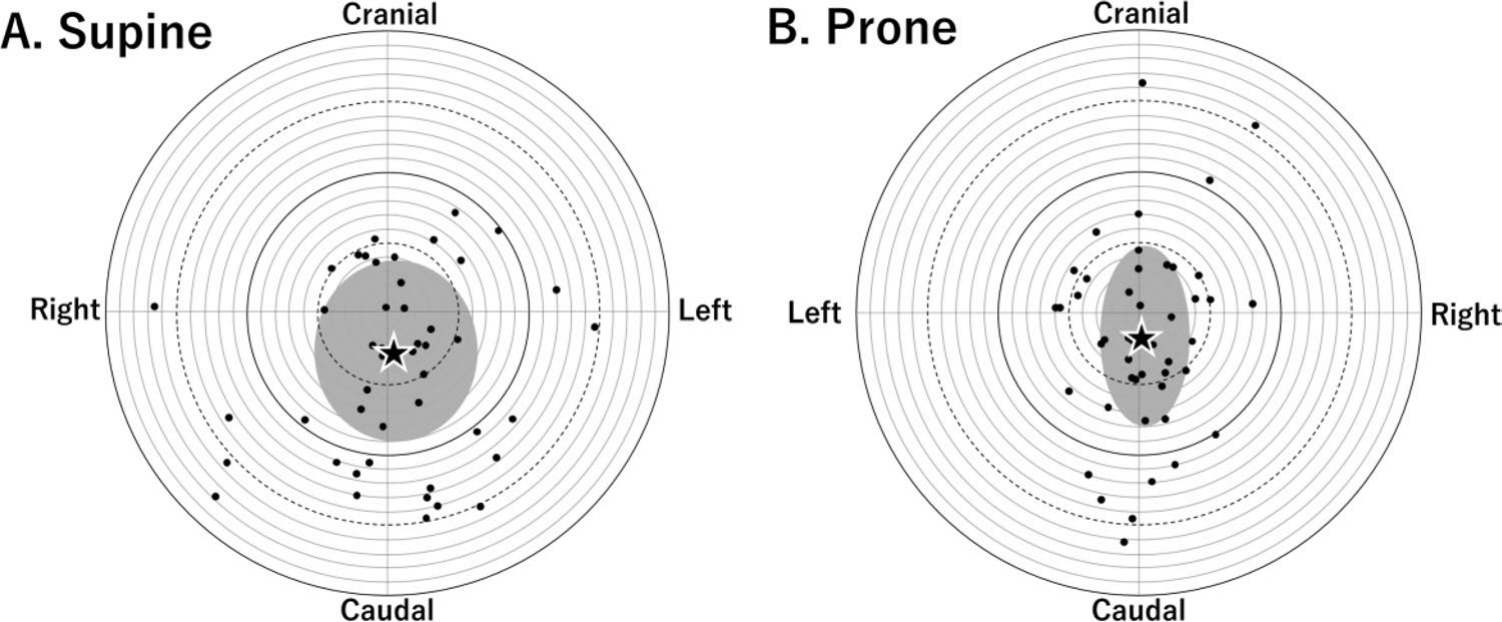
Landing points of the drone. (**A**) and (**B**) indicate the landing points of the drone in supine and prone positions, respectively. The center of the circles represents the umbilicus. The distance between a circle and the next circles is 1 cm. The black dots denote the individual landing points. The black stars denote the average landing position with the gray areas indicating the standard deviation.

The pilot successfully landed the drone on the target point on all the participants in both the supine and prone positions. The average landing point in the supine (Fig. 3A, left: 0.77 ± 5.8 cm, caudal: 3.2 ± 6.4 cm) and prone positions (Fig. 3B, right: 0.45 ± 3.3 cm, caudal: 1.7 ± 6.6 cm) were slightly caudal (Fig. 3). The landing points in the supine position seemed to be wider laterally than those in the prone position. The mean angles of the drone’s nose were − 1.9 ± 9.5 degree and − 0.7 ± 4.1 degree in the supine and prone positions, respectively.

## Discussion

This study demonstrated the following: (1) breathing was accurately evaluated when the drone was landed on the participants’ bodies compared to when it was kept hovering over them, (2) the number of correct answers in both the “breathing” and “no breathing” assessments was small when the drone was kept hovering than when it was landed on the participants’ bodies, (3) about 30% in incorrect answer was determined as completely incorrect (false negative) in both the supine and prone positions when the drone was kept hovering, and (4) the drone was successfully landed on the participants’ body in all the experiments, and the landing points seemed to be wider laterally in the supine position.

The strengths inherent to drones and their usage in public health have been identified^9^. The use of drones enhances the delivery of healthcare by providing faster response times, reduced transportation costs, and improved medical products/services to remote and/or underserved environments^10^. In fact, automated external defibrillators, blood products, organs, and medications have been transported using drones^10–12^. Drones can be used to transport automated defibrillators to the site of an out‑of‑hospital cardiac arrest within a short time and thus, increase the survival chances of such patients^13^.

Drones of various sizes have been successfully utilized to rescue disaster victims. However, no study has used drones to evaluate the breathing status of victims in small spaces. This is the first study to evaluate breathing status using a drone that directly landed on people’s bodies and hovered over them. Wide spaces allow most drones to perform rescue activities to the utmost degree. Indeed, the respiration signals can be reportedly extracted well with a low signal-to-noise ratio at a distance of 7 m using the 24 GHz Doppler radar system^14^. On the other hand, small confined spaces such as underneath debris and buildings impede drone activities. The mobility of small drones might change rescue operations in local and global disasters. In addition, several small drones might simultaneously evaluate multiple victims effectively. On the contrary, a disadvantage with the use of small drones is the issue with batteries. They are operated using a small-sized battery that provides shorter flight time; thus, the time of activity is restricted, resulting in limited detection of victims and insufficient evaluation of vital signs. Hence, there is a need for larger drones that transport several small drones to unsafe sites, called “mother drones.” This will resolve the issue of low battery capacity especially in cold sites where the batteries are easily consumed.

It is technically easier to keep the drones hovering over victims than landing the drones on them; therefore, we will focus on hovering the drone over the victims during a disaster to evaluate their breathing. The stability system of hovering has already been shown in commercial situations, but it would not be possible to evaluate breathing sufficiently with a hovering drone. Indeed, the percentage of correct answers when the drone was hovering was low in both the “breathing” and “no breathing” status. Additionally, regardless of the position (supine or prone) that the victims were in, we were still unable to correctly determine their breathing status when the drone was kept hovering. Blowing wind further deteriorates the quality of the footage transmitted from the hovering drones.

Providing a completely incorrect response when the victim is breathing (false negative) could cause harm to survivors. Hence, “completely incorrect” answers should be avoided. Triage tools should err on the side of reducing under-triage (false negative), but this might also increase the risk of over-triage (false-positive). The acceptable under-triage rate is reported to be 5%, while the acceptable over-triage rates may be as high as 50%^15^. However, we ought to also minimize the loss of time in attending to other survivors when we over-triage victims. As time passes, the rate of preventable deaths increases, especially for survivors with trauma^16–18^. Assessment when the drone was kept hovering did not minimize the frequency of under-triage and over-triage. Consequently, we cannot adopt the hovering method in triage scenes relative to the landing one.

Landing on victims is likely applicable to disaster situations. Notably, the evaluators were able to correctly determine the breathing of all the participants in the supine position, while the breathing in two participants was incorrectly determined in the prone position. The evaluators were able to easily evaluate breathing from the drone camera because the synergy between the chest motion and abdominal motion did not blur the images. Once a drone lands on the victims’ body, the focus is no longer on controlling the device and the consumption of battery power but on the evaluation of breathing status. Even in dusty and poorly visible conditions, the evaluators could still determine the breathing status as the drone can stay close to the victim’s bodies and has a stable visual field. In addition, the drone, weighing only 80 g, was not heavy on them. In the future, if drones were equipped with artificial intelligence, they automatically could detect victims, land on them, and judge the breathing condition.

Triage scenes, during mass casualty incidents capable of overwhelming healthcare resources, require responders to simply and swiftly judge the victims’ status, especially their breathing; thus, unclear assessments must be avoided. Taking our situation, we only provided the two options “breathing” and “no breathing,” and an “unclear” option was not included. However, breathing may take other forms such as agonal gasping breathing. We were unable to distinguish gasping from normal breathing using drones. Gasping is a type of breathing that requires the assistance of emergency medical services to rescue survivors. Gasping has been independently associated with a favorable neurological outcome^19^. Nevertheless, gasping is not easily recognized by inexperienced responders and healthcare workers^20–22^. In such settings, footage transmitted from a landed drone may allow the responders to closely and clearly examine the victim and to adequately assess the form of breathing. We need to evaluate how sensitive is this technology in the case of shallow or irregular breathing.

The evaluators had the anterior view through the drone camera in this study, but the drone landing was successfully performed in all experiments. Moreover, the angle of the drone’s nose was almost parallel to the cranial-caudal body axis. The pilots were trained to perform resuscitation using manikins at a fairly low cost. Medical staff can also be trained to land drones on human bodies through visual media. The successful landing of drones on victims’ bodies may depend on the shape of the abdomen or back. A rounded abdomen is unsuitable for landing, and the drone would need to be secured as in this study. Our drone could land on the abdomen and back of overweight participants with a body mass index of 30 kg/m^2^. Since we used a sticky skid to secure the drone in place, takeoff was difficult. To counter this, a mild sticky seal such as adhesive notepads can be used. Ultimately, takeoff after landing on the body may be unnecessary as small drones are not very expensive and can be disposable, and rescuers could collect them when they locate the victims. When the drone landed on a participant’s body in this study, the wide deviation observed when they were in the supine position may be due to the central area being at a higher level than the lateral area in the abdomen. Conversely, the narrow deviation observed while in the prone position may be due to the central portion of the back being at a lower level than the lateral portion.

In terms of safety, the propeller guards or/and collision protection systems help avoid preventable accidents. The propeller guards allow the drone to move while preventing it from hitting objects when it is operated at low speeds. Furthermore, the collision protection system under the drone prevents the drone from accidentally coming into contact with participants. In an actual triage setting, victims might move and dispel drones using their hands, when drones come close to them. The small drone is fragile and has a lesser collision risk. Next-generation commercial drones have all-dimensions protection systems that can be used safely for rescuing victims and are not difficult to operate.

## Limitations

The order of the supine and the prone position was not randomized in this study, leading to a slight bias of the judgment.

Low Wi-Fi connectivity (2.4 GHz 802.11n) restricts the drone’s activity in some disaster fields. Wi-Fi connectivity weakens due to obstacles made of various materials. The maximum transmitted distance from the controller to the drone was 100 m without any obstacles. This is a very important drawback. Hence, we hope that the Wi-Fi connectivity system can be improved in the future. Besides, we also need to check the durability of the Wi-Fi capability and the drone itself in fire, wind, smoke, dust, radioactive or electromagnetic radiation.

The lateral position is yet to be evaluated. No study has evaluated how victims lie on the ground in disaster scenes. Prior to this study, we conducted a preliminary study with the participants lying on their side; similar to this study, the participants’ breathing was evaluated through real-time footage of the lateral abdomen transmitted from the drone. The two evaluators were able to determine the breathing correctly in all 10 cases. This finding suggests that the breathing status of victims in a lateral decubitus position as well as in the supine position can be accurately evaluated. Breathing status should also be assessed in various positions. Moreover, one may argue that breathing movement may vary among participants. The complexity of not only chest movement but abdominal one is important to evaluate breathing. It is challenging that the movement is quantitatively verified. In addition, victims’ chest motion is poorly visible in the conditions such as dusty or dark places. However, we hope a small light or other devices attached to the drone will likely overcome that situation.

## Conclusion

Breathing was more reliably evaluated with a small drone landing on victim’s bodies than when the drone was kept hovering over them.
