# Author Correction: Real-time breath recognition by movies from a small drone landing on victim’s bodies

**DOI:** 10.1038/s41598-021-99295-9

**Published:** 2021-09-29

**Authors:** Takeji Saitoh, Yoshiaki Takahashi, Hisae Minami, Yukako Nakashima, Shuhei Aramaki, Yuki Mihara, Takamasa Iwakura, Keiichi Odagiri, Yuichiro Maekawa, Atsuto Yoshino

**Affiliations:** 1 Department of Emergency and Disaster Medicine, Hamamatsu University School of Medicine, Hamamatsu, 431-3125 Japan; 2 Center for Clinical Research, Hamamatsu University Hospital, Hamamatsu, 431-3125 Japan; 3 Department of Cardiology, Hamamatsu University School of Medicine, Hamamatsu, 431-3125 Japan

Correction to: *Scientific Reports* 10.1038/s41598-021-84575-1, published online 03 March 2021

The original version of this Article contained an error in the spelling of the author Keiichi Odagiri which was incorrectly given as Keiichiro Odagiri. The original Article has been corrected.

